# Anesthetic management for resection of para-aortic paraganglioma and unexpected aortic resection: A case report

**DOI:** 10.3892/etm.2015.2289

**Published:** 2015-02-13

**Authors:** CHERYL WANG, ROBERT RICHMOND, ENAS ELDESOUKI

**Affiliations:** 1 School of Medicine, University of Pittsburgh, Pittsburgh, PA 15213, USA; 2 VA Western New York Healthcare System, Buffalo, NY 14215, USA

**Keywords:** anesthesia, para-aortic paraganglioma, resection, case report

## Abstract

Paragangliomas account for 15–20% of pheochromocytomas derived from chromaffin cells and secretes catecholamines. It has a high mortality rate due to hypertension and challenging anesthetic management. The present report is of a case of the successful management of paraganglioma resection with unexpected aortic resection. The patient presented for paraganglioma resection. The blood pressure (BP) was well controlled with α blockade followed by β blockade prior to surgery. The patient was under general anesthesia, with multiple intravenous lines, catheters and an arterial line. Induction was achieved by the administration of narcotic and volatile agents. During the procedure, the aorta was found to require resection in order to complete the tumor resection. The BP changed markedly with clamping and unclamping, tumor vein ligation and tumor resection. The increased BP due to catecholamine release and unclamping was controlled with phentolamine, nitroprusside, esmolol and labetolol. Drops in BP due to tumor vein ligation and clamping were managed with norepinephrine and vasopressin. With close communication and monitoring, the surgery on the patient was successfully completed and the patient was discharged days later in a hemodynamically stable condition. The diagnosis was further confirmed by pathology. This was a challenging case of paraganglioma resection with unexpected aortic resection. The success achieved suggests that the resection of paraganglioma and an aortic segment requires delicate anesthetic management. The key are α blockade and β blockade as necessary to control BP pre-operatively, frequent communication between the anesthesiologist and surgeons, intra-operative intervention in excess catecholamine release with phentolamine, nitroprusside and labetalol prior to tumor removal, and vasopressin for catecholamine deficiency when clamping or subsequent to tumor removal. It is a delicately orchestrated process requiring team work.

## Introduction

Pheochromocytomas are derived from chromaffin cells and secrete catecholamines; 15–20% of pheochromocytomas are extra-adrenal and termed as paragangliomas (1). A high incidence of malignancy (13–26%) has been reported in paragangalioma (2). Complete surgical resection has been recommended as the mainstay of management (3). Yet, due to severe hypertension and its consequences, the anesthetic management has been quite challenging and the mortality rate remains high, particularly in those close to the aorta or in patients having aortic complications (4). Preoperatively, it is difficult to control blood pressure due to its pulse release of catecholamines and waves of blood pressure changes. Intraoperatively, there are fluctuations in blood pressure due to the clamping, maneuver, ligation of the arteries, lack of communication between the surgeons and anesthesiologist, and the dosing of the medications. Postoperatively, the hemodynamic state of the patient requires intensive monitoring. The present case report describes the successful anesthetic management used in a unique case of para-aortic ganglioma resection with unexpected aortic segment resection. The key factors cosnidered are α and β blockades as necessary to control BP preoperatively, frequent communication between the anesthesiologist and surgeon, intraoperative intervention in excess catecholamine release with phentolamine, nitroprusside and labetalol prior to tumor removal, and vasopressin for catecholamine deficiency when clamping or subsequent to tumor removal.

Multiple attempts have been made to contact the patient or their legal designee for consent; however, these have not been successful. Approval was thus sought from the VA Western New York Healthcare System Institutional Review Board (Buffalo, NY, USA) who determined that approval was not required.

## Case report

### Patient

A 64-year-old male was admitted to the VA Western New York Healthcare System for the resection of pheochromocytoma/paraganglioma. The patient had been diagnosed with pheochromocytoma during a previous surgery. This was further confirmed by the patient’s significant symptoms, magnetic resonance imaging (MRI; 5 mm lesion, 1.8 cm proximal to aortic bifurcation) and 24 h urine normetanephrine (4-fold greater than the normal upper limit) and vanillylmandelic acid (VMA; 1.5-fold greater than the normal upper limit). Systemic review revealed hypertension, hyperlipidemia, osteoporosis, rectal carcinoma and pheochromocytoma. The patient weighed 84 kg and was 168 cm in height.

### Pre-operative assessment and preparation

The patient had been taking an α-blocker (phenoxybenzamine, 10 mg twice per day, orally) for over a month and then a β-blocker (metoprolol). The blood pressure (BP) was maintained at ~120/80 mmHg, and the heart rate (HR) was 55 bpm. The Mallampati class was 2, the American Society of Anesthesiologists (ASA) class was 3, the hemoglobin level was 12.9 g/dl and the hematocrit was 38.3%. An electrocardiogram revealed no abnormalities and chest X-ray indicated no active disease. During the arterial line placement, the patient complained of nausea, the BP dropped to 80/50 mmHg and the HR rose to 70 bpm. This was resolved after the patient lay flat and a 500-ml bolus of normal saline was given.

### Intra-operative management

**Figure 1:**
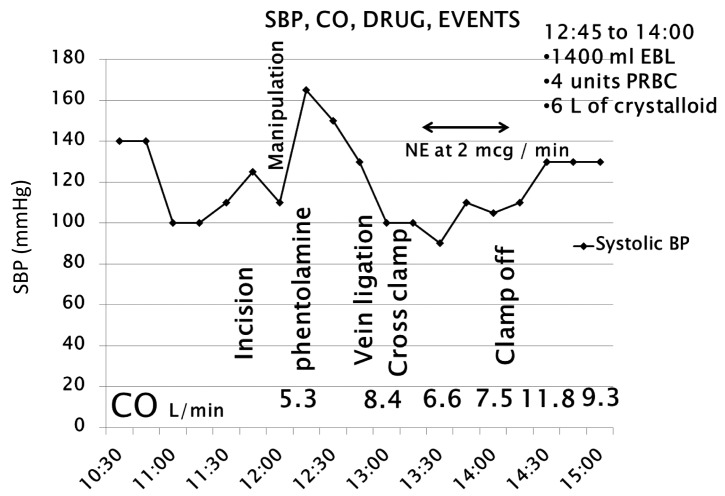
Intra-operative anesthetic management for the resection of paraganglioma and aortic segment in the patient. It shows that the CO and SBP changed with manipulation and clamping/unclamping of the aorta. The SBP was controlled within a reasonable range (85–135 mmHg) under the continuous infusion of NE at 2 μg/min when clamping/unclamping or after tumor removal. SBP, systolic blood pressure; CO, cardiac output; NE, norepinephrine; EBL, estimated blood loss; PRBC, packed red blood cells.

**Figure 2:**
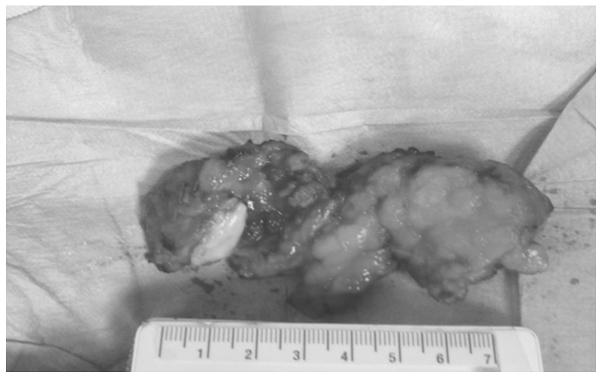
Gross mass of the resected paraganglioma and aortic segment.

The patient was placed under general anesthesia, with two large peripheral intravenous lines (PIVs), one arterial line, one right internal jugular central line and a pulmonary artery catheter. A total of 1–3 mg/kg/h propofol and 2–20 mcg/kg/dose fentanyl were used for induction, and nitroglycerine was readily available. During the procedure, aortic segment resection was required to complete the tumor resection. As shown in Fig. 1, due to tumor manipulation and catecholamine release, the BP of the patient increased. This was managed with phentolamine, nitroprusside, esmolol and labetolol. Tumor vein ligation and clamping triggered a reduction of the BP, which was managed with norepinephrine (NE) and vasopressin. Continuous NE infusion was set at a rate of 2 μg/min. During the whole procedure, the cardiac output was maintained within the normal range, 6 liters of crystalloid and 4 units of packed red blood cells were administered, and there was an estimated blood loss of 1,400 ml. The blood glucose level was 174 mg/dl. Fig. 2 shows the gross resected mass. Diagnosis was further confirmed by pathological examination, which identified an extra-adrenal paraganglioma, 4.3 cm in diameter, within a para-aortic ganglion and invading the aortic adventitia, without evidence of adrenal parenchyma or angiolymphatic invasion. Positivity for chromogranin and S100, a low proliferative index and negative nodes were observed.

### Post-operative management

Following the procedure, the patient was transferred to the intensive care unit (ICU) without pressor medication, intubated and hemodynamically stable. The patient was extubated the next morning, transferred to the ward after 24 h, and discharged 5 days later, in a hemodynamically stable condition.

## Discussion

To the best of our knowledge, this is the first case report of successful anesthetic management in the resection of paraganglioma along with unexpected aortic resection. The success achieved suggests that delicate management can reduce mortality, even with unexpected challenges. The key is maintaining the BP within a reasonable range and keeping the patient hemodynamically stable. The hallmarks include preoperative α blockade, and intraoperative intervention in excess catecholamine release prior to tumor removal and catecholamine deficiency following tumor removal.

As shown in Table I, we recommend that pre-operative management should focus on complete α blockade and β blockade as necessary to avoid hypertensive crisis during induction. A course of 10–14 days α blockade is recommended prior to the addition of β blockade. Notably, β blockade should never be initiated without successful α blockade, as this may trigger hypertensive crisis due to unopposed α-adrenergic receptor stimulation (5). A norepinephrine synthesis inhibitor may be used as an alternative. However, it generally takes 3 weeks to take effect. The common agents used are phenoxybenzamine, propanolol and metyrosine, respectively (3,6). Calcium channel blockers have also been used to achieve a better control of BP in addition to α blockade, for patients intolerant to α blockade, and those with intermittent hypertension (3,7). In the present case, the BP of the patient was well controlled with α blockade and β blockade as necessary pre-operatively. However, the patient complained of feeling unwell and BP changes occurred during induction, which were resolved by the patient lying down and receiving fluid resuscitation. This is a reminder that caution should be paid even with good BP control pre-operatively, as any stimulation of the vessels/tumor could dramatically change the vessel tone and thus BP.

For intra-operative management, frequent communication between surgeons and the anesthesiologist is extremely important, as manipulation of the tumor, clamping and unclamping of the aorta, and other stimulation can change the BP significantly. Increased BP could increase bleeding from small vessels. Due to catecholamine release, hyperglycemia presented during the operation. However, the over-treatment of hyperglycemia may cause significant post-operative hypoglycemia. It is recommended that an arterial line should be put in place prior to induction to monitor the BP. A central venous catheter and large-bore peripheral catheter should be used to manage resuscitation. The successful management in the present case suggests that changes in BP can be well controlled during surgery, based on pathophysiology. In the present case, α blockade was used to intervene in the excess catecholamine release due to tumor manipulation; nitroprusside and labetolol were also used to better control the BP, and NE was administered to maintain the BP which fell due to catecholamine deficiency when clamping or following tumor removal. This is consistent with the majority of studies (3,7), indicating that dedicated monitoring and intervention can benefit the patient and reduce mortality significantly. Magnesium and clevidipine have also been used to control or maintain BP (1).

It is recommended that post-operative management should be focused on blood glucose monitoring, BP normalization, urine 24 h catecholamine measurements 1–2 weeks after surgery and associated check-ups (6).

Certain food and drugs should be avoided or used with caution prior to surgery (5,6). Foods rich in tyrosine such as aged cheese, yogurt, sour cream, wine, beer, chocolate, smoked meats, fermented soy bean or fish products, nuts and certain fruits and vegetables should be avoided or limited in intake (5). Some drugs, such as β-blockers prior to α-blockers, dopamine D2 receptor antagonists, serotonin receptor inhibitors, NE receptor inhibitors, monoamine oxidase inhibitors, tricyclic antidepressants, sympathomimetics, chemotherapeutic agents, opiates, neuromuscular blockers, peptides and steroids should also be avoided (3).

In conclusion, the anesthetic management in this challenging case of para-aortic ganglioma resection with unexpected aortic segment resection was successful. It suggests that resection of a paraganglioma and aortic segment requires delicate anesthetic management. The key features are α blockade and β blockade as necessary to control the BP pre-operatively, frequent communication between the anesthesiologist and surgeons, intra-operative intervention in excessive catecholamine release with phentolamine, nitroprusside and labetalol prior to tumor removal, and vasopressin for catecholamine deficiency when clamping or subsequent to tumor removal. It is a delicate procedure, requiring teamwork and orchestration.

## Figures and Tables

**Table I:** Management of para-ganglioma resection.

Management	Recommendation
Operative	
Pre-operative	α-blocker (≥2 weeks), β-blocker (after α-blocker), cathecholamine synthesis inhibitor, calcium channel blocker
Intra-operative	Communication between surgeons, nitrupresside/labetolol for BP, NE for tumor vein ligation/clamping, magnesium, clevidipine
Post-operative	Vasopressin, blood glucose, urine VMA
Avoid/caution	
Food	Aged cheese, yogurt, sour cream, wine, beer, chocolate, smoked meats, fermented bean or fish products, nuts, certain fruits and vegetables
Drugs	β blockade before α blockade, D2 receptor antagonists, serotonin/NE receptor inhibitors, sympathomimetics, chemotherapeutics, α-blocker for tumor manipulation, opiates, neuromuscular blockers, peptides and steroids

Food and drugs should be avoided or used with caution mainly for peri-operative management. BP, blood pressure; MAO, monoamine oxidase inhibitor; TCA, tricyclic antidepressant; VMA, vanillylmandelic acid; NE, norepinephrine; D2, dopamine 2.
